# Clinical course and significance of nontuberculous mycobacteria and its subtypes in cystic fibrosis

**DOI:** 10.1186/s12879-018-3200-z

**Published:** 2018-07-06

**Authors:** Maxine S. Eikani, Melodee Nugent, Arash Poursina, Pippa Simpson, Hara Levy

**Affiliations:** 1 Novant Health Pediatric Pulmonology, Novant Health Hemby Children’s Hospital, Charlotte, NC USA; 2 Children’s Research Institute, Children’s Hospital of Wisconsin, Milwaukee, WI USA; 3 Quantitative Health Sciences, Medical College of Wisconsin, Milwaukee, WI USA; 4 Piedmont Medical Center Infectious Disease, Piedmont Medical Center, Rock Hill, SC USA; 5 Division of Pulmonary, MedicineNorthwestern University Feinberg School of Medicine, Ann and Robert H. Lurie Children’s Hospital, Chicago, IL USA; 6 Department of Pediatrics, Section of Pulmonary Medicine, Human Molecular Genetics Program, Stanley Manne Children’s Research Institute, Northwestern University Feinberg School of Medicine, 225 E. Chicago Ave, Chicago, IL USA

**Keywords:** Cystic fibrosis, Nontuberculous mycobacteria, Infectious disease, Pulmonary function test, Forced expiratory volume

## Abstract

**Background:**

Nontuberculous mycobacteria (NTM) infections in patients with cystic fibrosis (CF) is increasing globally. However, the related epidemiology, comorbidities, and clinical impact of NTM infection remains unclear in the progress of CF lung disease and patient survival.

**Methods:**

We performed a retrospective, case-control, cohort study (10 years), comparing NTM culture-positive CF patients (*N* = 28) to matched controls (*N* = 26). NTM positive patients were divided in to two groups of slow-growing (*N* = 17) and rapid- growing NTM (*N* = 8). Three patients were positive for both slow and rapid NTM. For independent group comparisons, a non-parametric Mann-Whitney test (Kruskal-Wallis test for more than two groups) was used to compare the continuous variables, and a Fisher’s exact test was used for the categorical variables. Paired comparisons were performed using a Wilcoxon signed-rank test.

**Results:**

The prevalence of NTM isolation was 8%. The age at CF diagnosis was significantly lower in the slow-growing NTM group compared to the rapidly growing NTM group (*P* = 0.04). The median percent predicted forced expiratory flow of 25% − 75% (FEF25–75) was significantly higher before NTM acquisition in slow-growing (*P* = 0.013) and rapidly growing NTM group (*P* = 0.028). The slow-growing NTM group received significantly more penicillin/beta lactamase (*P* = 0.010) and rifampin (*P* = 0.042) following isolation. Macrolide use was significantly higher after isolation in both the slow-growing NTM (*P* = 0.018) and rapidly growing NTM groups (*P* = 0.042).

**Conclusions:**

An earlier CF diagnosis was associated with a higher isolation of slow-growing NTM and greater antimicrobial use after infection. NTM acquisition is associated with a worsening of FEF25–75. Thus, both the early diagnosis and treatment of an NTM infection in patients with CF may positively impact lung function.

## Background

Cystic fibrosis (CF) is the most common life-limiting autosomal recessive disease of Caucasians in the United States [1]. Improvement in the life expectancy of patients with CF is complicated by potential infection with highly resistant bacterial strains [1, 2], newly recognized virulent pathogens, and organisms, such as nontuberculous mycobacteria (NTM), which have an undetermined significance of infection associated with different subspecies [2–5]. NTM are a group of heterogeneous environmental organisms found in the soil and water throughout the world; these bacteria have historically been broadly classified into “slow” and “rapid” growers [6]. In addition, NTM may be associated with sample contamination and a spectrum of conditions ranging from asymptomatic infection to severe symptomatic disease [6]. Moreover, the estimated prevalence of NTM infection in patients with CF appears to be increasing (from 1.3% in 1984 to 32.7% in more recent studies) [3–8]. This increase in prevalence could be due to improvement in CF patient survival, more accurate laboratory detection techniques, and heightened clinician awareness of NTM-related lung disease [4]. Although the recognition and diagnosis of NTM-related lung diseases seems to have improved, likely due to increased awareness, consistent testing and reliable and effective treatment of NTM, especially in patients with CF, remains problematic.

NTM species diversity within CF patient populations appears to vary with geography [6, 7]. The pulmonary NTM pathogens that are most commonly isolated from CF patients in North America include the *Mycobacterium avium* complex (MAC), consisting of slow-growing bacteria, and the *M. abscessus* complex (MABSC), consisting of rapid growers [7, 9]. In contrast, MABSC is isolated from CF patients more frequently than MAC in Western Europe and Israel [7, 10–16].

Attempts to identify risk factors that can be used to predict the development of NTM infection in CF patients have not yielded consistent results. In several studies, NTM-positive CF individuals appear to be older than their NTM culture-negative counterparts [4, 7, 14, 15, 17]. Moreover, respiratory infection with MAC seems to have only a small effect on the health of CF patients [16]. In contrast, MABSC-positive patients frequently exhibit severe and occasionally fatal, lung disease [10, 18, 19]. Lung function, as assessed by percent of predicted forced expiratory volume in 1 second (FEV1), has been reported to have variable associations with NTM infection [4, 7, 10, 14, 17, 20]. In addition, the role of coexisting microbial pathogens and drug exposure remain poorly understood in the development of NTM infection. For example, *Aspergillus fumigatus* seems to be more prevalent in certain NTM-positive patients [8, 14, 17, 21, 22].

Treatment for NTM can be a lengthy and arduous process that is complicated by significant adverse effects [23]. Thus, a timely and accurate diagnosis of progressive NTM disease followed by appropriate management is likely to positively impact the long-term mortality and morbidity associated with CF and its treatment [7]. To meet these goals, this study sought to: 1) describe the prevalence of NTM in a large urban tertiary care university hospital and CF center; 2) characterize epidemiological factors (e.g., age, genotype, pulmonary function, co-infections, and CF comorbidities) associated with the acquisition of NTM and specific complexes; and 3) monitor the changes in antimicrobial treatment and patient pulmonary function following NTM infection.

## Methods

### Study patients

This retrospective, case-control, cohort study matched NTM culture-negative CF patients to culture-positive patients with CF based on genotype, age, and gender at the Children’s Hospital of Wisconsin Cystic Fibrosis Center (CHW, Milwaukee, WI). This study was approved by the Children’s Hospital of Wisconsin (CHW) Institutional Review Board (518358–8). Patients or their guardians provided informed written consent to access de-identified records from the CF Foundation registry and CHW to be used for research purposes. Clinical and research databases were queried to identify all patients diagnosed with CF based on the accepted criteria [24] who received standard care that included a minimum of quarterly visits to the CHW from December 2003 to December 2013, as well as a sample for sputum/bronchoalveolar lavage-based microbiological culture.

We included patients with an established diagnosis of CF and at least one positive NTM culture from a sputum and/or bronchoalveolar lavage sample. The exclusion criteria were defined as no history of CF and patients with CF whose acid fact bacteria (AFB) cultures were positive prior to the study period [16]. We initially identified patients with positive NTM cultures by querying the CHW Microbiology Lab database. All available patient records were reviewed to confirm the dates and details of the positive cultures. The medical records of patients meeting the inclusion criteria (*N* = 30) were reviewed from 1 year before the positive NTM culture until 1 year after the last positive culture or the end of the study period. Patients with positive NTM cultures were divided solely based on the isolated NTM species (slow growing and rapid growing). Acute, chronic, or transient NTM infection was not identified. All data were obtained from the patients’ medical records and the CF Foundation data registry.

For comparison purposes, one NTM culture-negative patient was matched to each NTM culture-positive patient based on CF mutation (by class) [25], age, and gender. Due to the limited number of culture-negative patients, four culture-negative patients were repeatedly matched to culture-positive patients. The timeline of the recorded data for each culture-negative patient was similar to the matched culture-positive patient.

### Data collection

Demographic, clinical, and laboratory data for all eligible patients were collected from the CF Foundation registry and CHW medical records. These data included age, age at CF diagnosis, gender, race, method of CF diagnosis, CF genotype, sweat-chloride levels, pancreatic function (fecal elastase < 200 μg/g indicating pancreatic insufficiency) [26], weight, height, body mass index, FEV1, forced expiratory flow at 25% − 75% predicted of the pulmonary volume (FEF25–75), baseline bacterial colonization, history of allergic bronchopulmonary aspergillosis (ABPA) [27], history of diabetes (type 1, type 2, or CF-related) [28], chronic medication use (macrolide, systemic steroid, inhaled steroid, inhaled antimicrobial, and inhaled hypertonic saline), serum IgE levels, serum vitamin levels (A, E, and D), NTM species identified from culture, whether the patient met the American Thoracic Society/Infectious Diseases Society of America criteria for an NTM infection [6], length and method of treatment for NTM (if any), and associated imaging changes (chest x-ray and computed tomography).

Methods of CF diagnosis were determined from the CF Foundation data registry; these methods included newborn screening and evaluation of concomitant symptoms (e.g., meconium ileus, family history of CF, recurrent sinopulmonary infections, failure to thrive, greasy or bulky stools, nasal polyps, and other signs) [24]. Patient medical records were reviewed for a history of type 1, type 2, and CF-related diabetes. CF-related diabetes was defined as oral glucose tolerance ≥200 mg/dL (≥ 11.1 mmol/L) and/or fasting plasma glucose levels ≥126 mg/dL (≥ 7.0 mmol/L) [29]. The diagnosis of ABPA was based on the presence of clinical symptoms, recent changes in chest imaging, serum total IgE levels > 1000 IU/mL (> 2400 ng/mL), immediate cutaneous reactivity to *Aspergillus* or in vitro presence of serum IgE and IgG antibodies to *A. fumigatus* [6]. FEV1 and FEF25–75 % predicted were expressed as the percent of predicted using the reference equations of Hankinson et al. [10]. Mycobacterial cultures were performed by the CHW Microbiology Lab using standard protocols to enhance the recovery of pathogens from the respiratory secretions of CF patients [7].

### Data management

The study data were collected and managed using Research Electronic Data Capture (REDCap) tools hosted at Medical College of Wisconsin. This system is a secure, web-based application designed to support data capture for research studies, providing: 1) an intuitive interface for validated data entry; 2) audit trails for tracking data manipulation and export procedures; 3) automated export procedures for seamless data downloads to common statistical packages; and 4) procedures for importing data from external sources. Drop-down menus and range checks were incorporated for quality control.

### Statistical analysis

SPSS version 22 (IBM Software, Chicago, IL, USA) was used to analyze the data. For independent group comparisons, a non-parametric Mann-Whitney test (Kruskal-Wallis test for more than two groups) was used to compare the continuous variables, and a Fisher’s exact test was used for the categorical variables. Paired comparisons were performed using a Wilcoxon signed-rank test. Percentage estimates are reported with their respective their respective 95% confidence intervals (CI) and continuous data with the median and range. *P*-values < 0.05 were considered significant.

## Results

Of the 360 patients with CF who received care at CHW during the study period, 30 (8%) were identified as having had at least one positive NTM culture from a sputum sample or bronchoalveolar lavage (95% CI: 5.7−11.7). Two patients were excluded from subsequent analysis because their sputum positivity predated the study period. All patients with CF who were included in the study (*N* = 28) visited the CHW CF care center at least four times per year. The total number of reviewed visits per patient ranged from 4 to 42 (median: 12; Fig. 1).

**Fig. 1:**
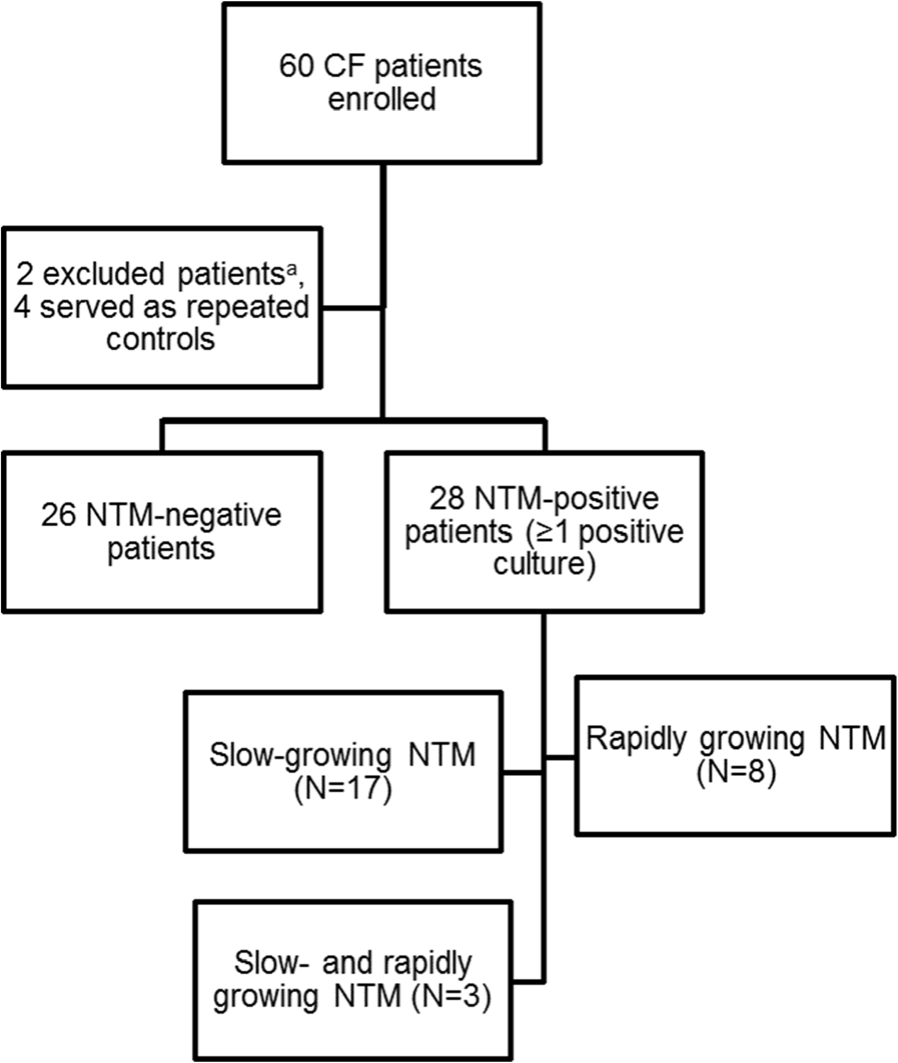
Selection process of the patient cohort included in this study. a Patients who were excluded because positive cultures were obtained before the study period

Of the 17 patients infected with slow-growing NTM, 15 (88% [95% CI: 63 − 99]) harbored MAC and two (12% [95% CI: 2 − 36]) harbored *M. gordonae.* Eight patients were infected with rapidly growing mycobacteria: six patients with MABSC (75%) and two patients (25%) with *M. fortuitum*. The median age at the time of the first NTM isolation was 16.4 years; patients infected with slow-growing NTM ranged in age from 6.4 to 41.6 years and patients infected with rapidly growing NTM ranged in age from 3.1 to 21.5 years (*P* = 0.44).

Although newborn screening for CF began in 1994 in Wisconsin and was implemented nationwide in 2010 [24, 30], variability remains for the age at diagnosis and missed cases of CF [30]. The age of CF diagnosis for those with slow-growing NTM was significantly lower (median: 1.2 months [range: 0.1 − 2.8 months]) compared to those with rapidly growing NTM (median: 4.5 months [range 1.5 − 84.3 months]) and NTM-negative patients (2.4 months [range: < 1 − 164 months]; *P* = 0.04; Table 1). The patients’ age at CF diagnosis significantly differed between those infected with rapidly growing NTM and the NTM-negative patients (*P* = 0.013).

**Table 1:** Patient demographics

	Patients with Slow-growing NTM #1 (*N* = 17)		Patients with Rapidly Growing NTM #2 (*N* = 8)		NTM-negative Patients #3 (*N* = 26)		*P*-values			
	N	*N* (%) or median (range)	N	*N* (%) or median (range)	N	*N* (%) or median (range)	Overall	1 vs 2	1 vs 3	2 vs 3
Age at first positive NTM culture, years	17	16.4 (6.4–41.6)	8	16.4 (3.1–21.5)	–		0.44^a^	0.44^a^		
Gender	17		8		26		0.93	0.99	0.75	0.99
Male		9 (53)		5 (63)		16 (62)				
Female		8 (47)		3 (37)		10 (38)				
Age at CF diagnosis in months	11	1.2 (0.1–2.8)	5	4.5 (1.5–84.3)	22	2.4 (< 1–164.4)	0.046	0.013	0.053	0.52
Race	15		8		26		0.37	0.35	0.29	0.99
Caucasian		15 (100)		7 (88)		23 (88)				
Black/Hispanic		–		1 (12)		3 (12)				
CF diagnosis method ^b^	12		5		24					
Newborn screening		8 (67)		1 (20)		10 (42)	0.24	0.18	0.75	0.23
Concomitant symptoms										
Meconium		3 (25)		–		5 (21)	0.51	0.53	0.99	0.31
Chronic cough		1 (8)		–		–	0.49	0.99	0.40	–
Recurrent respiratory infection		2 (17)		4 (80)		10 (42)	0.08	0.06	0.09	0.69
Failure to thrive		3 (25)		4 (80)		8 (33)	0.25	0.16	0.48	0.41
Frequent greasy or bulky stools		1 (8)		–		–	0.49	0.99	0.40	–
Nasal polyps		–		–		1 (4)	0.92	–	0.99	0.99
Other		1 (8)		2 (40)		4 (17)	0.41	0.23	0.63	0.61
p.F508del mutation status	17		8		26		0.44	0.46	0.25	0.84
2 copies		10 (59)		4 (50)		17 (65)				
1 copy		7 (41)		3 (38)		6 (23)				
0 copies		–		1 (12)		3 (12)				
Sweat-chloride level at diagnosis, mmol/L	13	101 (74–121)	5	105 (100–142)	22	109 (90–137)	0.026^c^	0.095	0.009	0.99
Pancreatic insufficiency	17		8		26		0.92	–	0.99	0.99
No		–		–		1 (4)				
Yes		17 (100)		8 (100)		25 (96)				
Diabetes diagnosis	17		8		26		0.67	0.99	0.67	0.57
No		14 (82)		6 (75)		23 (88)				
Yes		3 (18)		2 (25)		3 (12)				
FEV1 percent predicted										
Before NTM acquisition	17	92 (53–115)	7	84 (71–102)	–	–	0.80	0.80	–	–
After NTM acquisition	17	91 (36–110)	8	80 (48–91)	–	–	0.23	0.23	–	–
FEF25–75% predicted										
Before NTM acquisition	17	79 (24–124)	7	64 (42–123)	–	–	0.80	0.80	–	–
After NTM acquisition	17	68 (13–110)	8	51 (24–103)	–	–	0.59	0.59	–	–
Organism type	17		8		–		≤0.001	≤0.001	–	–
MAC		15 (88)		–		–				
*M. fortuitum*		–		2 (25)		–				
*M. abscessus*		–		6 (75)		–				
*M. gordonae*		2 (12)		–		–				
Number of positive NTM cultures	17	1 (1–3)	8	2.5 (1–7)	–	–	0.18	0.18	–	–

^a^Significant difference between patients infected with rapidly growing bacteria and NTM-negative patients (*P* = 0.013)

^b^More than one method of diagnosis may have been used

^c^Significant difference between patients infected with slow-growing bacteria and NTM-negative patients (*P* = 0.009)

Patient demographics, including gender, race, and method of CF diagnosis, did not significantly differ based on infection status (Table 1). The most common CF genotype in both groups was F508del homozygous (59% of patients infected with slow-growing NTM and 50% of patients infected with rapidly growing NTM; Table 1). The sweat-chloride levels significantly differed between those with slow-growing NTM (median: 101 mmol/L [range: 74 − 121 mmol/L]) and rapidly growing NTM (median: 105 mmol/L [range: 100 − 142 mmol/L]) and NTM-negative patients (median: 109 mmol/L [range: 90 − 137 mmol/L]; *P* = 0.026), as well as between the patients infected with slow-growing NTM and the NTM-negative patients (*P* = 0.009; Table 1). All patients in the slow- and rapidly growing NTM groups exhibited pancreatic insufficiency; only one patient in the NTM-negative group displayed pancreatic sufficiency (Table 1). There was no significant difference between the three groups regarding a history of diabetes or FEV1 before or after NTM acquisition (Table 1).

The FEF25–75 was evaluated to assess the potential effect of an NTM infection on the smaller airways. The median FEF25–75 (expressed as the percent predicted) before the acquisition of slow-growing NTM was 79 (range: 24 − 124) and after acquisition was 68 (range: 13 − 110; *P* = 0.013; Table 2). This difference was also significant for patients infected with rapidly growing NTM, who had a median FEF25–75% predicted of 64 (range: 42–123) before NTM acquisition and 51 (range: 24 − 103) after acquisition (*P* = 0.028; Table 2).

**Table 2:** Comparison of patients infected with slow-growing or rapidly growing NTM

	Patients with Slow-growing NTM (*N* = 17)			Patients with Rapidly Growing NTM (*N* = 8)		
	N	Median (range)	*P*-value	N	Median (range)	*P*-value
Body mass index in kg/m^2^	16		0.35	8		0.069
Before NTM infection		19.8 (15.0–23.9)			19.3 (14.7–21.7)	
After NTM infection		19.6 (15.2–25.9)			20.4 (15.1–21.7)	
FEV1 percent predicted	17		0.11	7		0.23
Before NTM infection		92 (53–115)			84 (71–102)	
After NTM infection		91 (36–110)			82 (48–91)	
FEF25–75% predicted	17		**0.013**	7		**0.028**
Before NTM infection		79 (24–124)			64 (42–123)	
After NTM infection		68 (13–110)			51 (24–103)	
IgE in kU/L	16		0.55	7		0.61
Before NTM infection		34 (8–2165)			95 (18–419)	
After NTM infection		34 (9–1340)			182 (57–429)	
Vitamin A in μg/dL	11		0.19	5		0.23
Before NTM infection		39 (19–83)			33 (15–45)	
After NTM infection		45 (25–79)			30 (12–41)	
Vitamin E in mg/L	9		0.37	5		0.50
Before NTM infection		8 (2–13)			7 (6–11)	
After NTM infection		9 (2–14)			5 (4–12)	

**Fig. 2:**
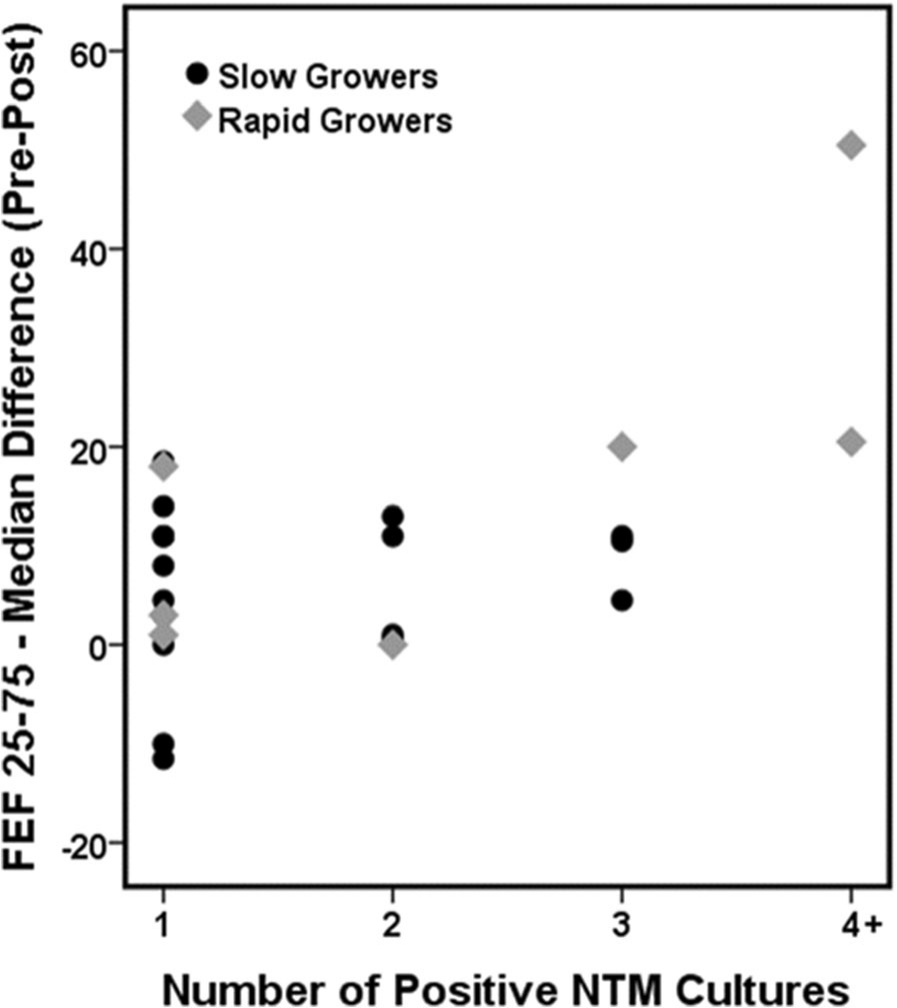
Median difference in the FEF 25–75 in CF patients with more than four positive cultures

Of the culture-positive patients, 13 (52%) had one positive culture and 15 (48%) had multiple positive cultures. CF patients with more than four positive cultures for rapidly growing NTM had a larger range for the median FEF25–75 (Fig. 2, Table 3), suggesting that the patients with more positive cultures had a lower FEF25–75 (Fig. 2; Table 3). The most common bacterial colonization in both infection groups was *Pseudomonas aeruginosa*, followed by *Staphylococcus aureus*, *Stenotrophomonas maltophilia*, and *Acinetobacter baumannii* (Table 4).

**Table 3:** Median difference in FEF25–75 before and after NTM acquisition vs. the number of positive cultures

	1	2	3	4
Slow growers				
N	10	4	3	0
Median	6.3	6.0	10.5	–
Range	−11.5-18.5	0.5–13.0	4.5–11.0	–
Rapid growers				
N	3	1	1	2
Median	3.0	0	20	35.5
Range	1.0–18.0	–	–	20.5–50.5

**Table 4:** Microbiology of the patient cohort

	Patients with Slow-growing NTM (*N* = 17)		Patients with Rapidly Growing NTM (*N* = 8)		
	N	N (%)	N	N (%)	*P*-value
Common bacterial colonization at time of positive NTM^a^	17		8		
*P. aeruginosa*		13 (76)		8 (100)	0.27
Methicillin-susceptible *S. aureus*		13 (76)		5 (63)	0.64
Methicillin-resistant *S. aureus*		6 (35)		3 (38)	0.99
*S. maltophilia*		4 (22)		4 (50)	0.36
*Escherichia coli*		1 (6)		–	0.99
*A. baumannii*		–		1 (13)	0.32
None		1 (6)		–	0.99
History of ABPA^b^					
Before NTM infection	17		8		0.53
No		14 (82)		8 (100)	
Yes		3 (18)		–	
After NTM infection	17		8		0.64
No		13 (76)		5 (62)	
Yes		4 (24)		3 (38)	
American Thoracic Society treatment criteria	16		6		
Clinical		11 (69)		6 (100)	0.27
New changes on chest x-ray or computed tomography		7 (44)		3 (50)	0.99
Two positive sputum or bronchoalveolar lavage		11 (69)		4 (67)	0.99
Received treatment for NTM	16		8		0.58
No		14 (88)		6 (75)	
Yes		2 (12)		2 (25)	

^a^Nontuberculous mycobacteria

^b^Allergic bronchopulmonary aspergillosis

Mycobacterial lung infection was assessed based on the American Thoracic Society criteria [9]. A total of 11 out of 16 (69%) patients infected with slow-growing NTM and all 6 (100%) patients infected with rapidly growing NTM exhibited clinical symptoms of an increased cough, sputum production, or shortness of breath at rest and upon exertion at the time of the positive NTM culture (Table 4). Seven patients (44%) infected with slow-growing NTM and three patients (50%) infected with rapidly growing NTM displayed image changes (e.g., new infiltrates, new bronchiectasis, consolidation, and nodules) on chest x-ray or computed tomography (Table 4). There were 11 patients infected with slow-growing NTM (69%) and 4 patients infected with rapidly growing NTM (67%) who had two positive sputum cultures or 1 positive bronchoalveolar lavage (Table 4). Only two patients infected with slow-growing NTM (12%) and two patients infected with rapidly growing NTM (25%) received treatment for NTM infection (Table 4).

Significantly more patients infected with slow-growing NTM received penicillin/beta-lactamase inhibitors following NTM acquisition (median: 4 [range: 0 − 9]) than those prior to NTM acquisition (median: 0.5 [range: 0 − 3]; *P* = 0.010). These patients also received rifampin following NTM acquisition (median: 5; [range: 2 − 7]); *P* = 0.042; Table 5). Macrolide use was significantly more frequent after NTM acquisition than before acquisition in both the slow-growing (*P* = 0.018) and rapidly growing NTM groups (P = 0.042; Table 5).

**Table 5:** Quantitative comparison of antimicrobial prescriptions before and after NTM acquisition

		Patients with Slow-growing NTM (*N* = 17)			Patients with Rapidly Growing NTM (*N* = 8)			
		Before	After			Before	After	
	N	Median (range)	Median (range)	*P*-value	N	Median (range)	Median (range)	*P*-value
Penicillins/Beta lactamase	12	0.5 (0–3)	4 (0–9)	**0.010**	4	1.5 (0.2)	1.5 (1.6)	0.65/0.41
Cephalosporin	5	2 (0–5)	1 (0–2)	0.99	2	1 (0–2)	13.5 (0–27)	0.66
Glycopeptide ^a^	–	–	–	–	1	0	2	–
Carbapenems	3	0 (0–0)	2 (1–8)	0.11	2	0.5 (0–1)	5.5 (0–11)	0.66
Monobactam ^b^	2	0.5 (0–1)	0.5 (0–1)	0.99	3	0 (0–0)	2 (1–3)	0.11
Aminoglycosides	11	1 (0–5)	3 (0–10)	0.20	5	2 (0–5)	5 (2–18)	0.18
Macrolides	7	0 (0–0)	5 (1–8)	**0.018**	5	0 (0–2)	3 (1–17)	**0.042**
Lincosamides ^c^	2	0 (0–0)	1 (1–1)	0.16	1	1	0	0.32
Oxazolidinones ^d^	1	0	1	0.32	1	0	1	0.32
Fluoroquinolones	9	2 (0–4)	2 (0–4)	0.99	6	0.5 (0–2)	4.5 (0–26)	0.12
Antifolates ^e^	8	1 (0–7)	1 (0–13)	0.40	4	1 (0–4)	2 (1–4)	0.41
Rifamycins ^f^	5	0 (0–0)	5 (2–7)	**0.042**	0	–	–	–

^a^Vancomycin

^b^Aztreonam

^c^Clindamycin

^d^Linezolid

^e^Trimethoprim/sulfonamides

^f^Rifampin

There were three culture-positive patients infected with both MAC and MABSC at different times during the study period. All three patients were Caucasian and one was male; they were aged 7, 15, and 18 years at the time of the positive NTM culture. Two patients were homozygous for F508del. One was colonized with *S. aureus*, and the other two were colonized with *S. aureus* and *P. aeruginosa*. The median FEV1 was 86, 104, and 98 before NTM acquisition, and 75, 74, and 100 after NTM acquisition. The median FEF25–75% predicted was 72, 91, and 86 before NTM acquisition and 54, 49, and 87 following NTM acquisition.

## Discussion

There has been an increase in the isolation of NTM bacteria from the respiratory secretions of CF patients [2–6]. Although NTM infection was present in 8.3% of our CF patients during the study period, this value is relatively similar to previous studies [2, 5, 8–11]; however, this may not reflect the true prevalence. Many of our CF patients rarely underwent mycobacterial analysis and some never underwent any mycobacterial testing. This omission may be due to difficulties with the collection of adequate and appropriate specimens in younger patients, the indeterminate significance of these organisms in the CF population, and a lack of awareness and consistency among providers in obtaining NTM cultures as part of routine CF care [7].

More than 160 species of NTM have been characterized to date; of which a select few are associated with clinical disease in humans. Each member of this heterogeneous group of organisms has its own microbiological and clinical significance, with equally diverse treatment and resistance profiles [6]. The most common NTM species isolated in our study was MAC, followed by MABSC (Table 1); this observation is consistent with other reports [2, 4, 7, 9, 10, 15, 17, 31]. Catherinot et al. [16] compared CF patients infected with MAC and MABSC in France and found that MAC was more common in adult patients with mild CF, whereas MABSC more frequently infected younger patients with more severe CF [16]. Our results do not fully confirm the results of Catherinot et al., since the median age was similar between the culture-positive groups (Table 1). Historically, *M. gordonae* was classified as the most common contaminant NTM species; however, there have been reports of infection with this organism in patients with CF [6, 18].

Our analyses revealed a significant relationship between the patients’ age at CF diagnosis and infection with rapidly growing mycobacteria (Table 1). Early CF diagnosis via newborn screening and recent advances in medical care are expected to facilitate better preventive care and management of CF patients infected with NTM [28]. Delays in the initial diagnosis of CF generally lead to a late start in patient management, potentially resulting in poorer general health, malnutrition, and more advanced lung disease, all of which contribute to infection with rapidly growing mycobacteria [28, 31].

Multiple prospective and retrospective studies have yielded inconsistent results regarding the possible effects of NTM infection on the progression of CF lung disease [7, 10, 14, 17, 20]. To the best of our knowledge, the current investigation is the first single center study in the US to compare the effects of slow- and rapidly-growing NTM on the smaller airways by comparing the FEF25–75 before and after NTM acquisition in CF patients. Previous studies have suggested that FEF25–75 is a sensitive indicator of early disease in children with CF [13], which our current study further supports. Similarly, Bakker et al. [13] reported that FEF75 is a more sensitive marker of early CF lung disease than FEV1 and forced vital capacity because abnormalities in FEF75 occur at a younger age and FEF75 decreases more than other pulmonary-function parameters. In the present study, FEV1 as a measure of pulmonary function did not differ before and after NTM acquisition in either infection group; however, we did detect significantly lower FEF25–75 after NTM acquisition in both culture-positive patient groups (Table 2). Patients with more than four cultures positive for rapidly growing NTM were associated with the greatest change in the median FEF25–75 throughout the follow-up period (Table 3; Fig. 2). This is likely because patients with rapidly growing NTM can have more acute and severe clinical symptoms [6, 7]. Our data suggest that NTM infection has a negative impact on small airway function in patients with CF.

Coexisting microbial pathogens have an undetermined role in the development of NTM infection. In the current study, the most common bacterial colonization in both culture-positive groups was *P. aeruginosa*, followed by *S. aureus* (Table 4). A variable prevalence of NTM isolation in patients with underlying *P. aeruginosa* and *S. aureus* colonization has been reported in multiple studies [10, 14]; however, there is some evidence of a higher prevalence of *A. fumigatus* colonization among NTM-positive patients [10, 14, 16], as well as an association of NTM with ABPA [22]. Despite these previous reports, we failed to detect a significant relationship between a history of ABPA and NTM acquisition.

Penicillin, beta-lactamase inhibitors, and rifampin use was higher in our patients infected with slow-growing NTM compared to the patients infected with rapidly growing NTM (Table 5). This observation may reflect a decline in patient clinical status that leads to more frequent hospitalization and antibiotic administration in inpatient and ambulatory settings. Many of the antimicrobials used to treat underlying bacterial colonization exhibit some activity against NTM species and may interfere with an accurate diagnosis of the infection and evaluation of subsequent pulmonary function [6, 7]. Unfortunately, inadequate and inappropriate exposure of NTM to antimicrobials can also lead to the development of antibiotic resistance [32].

The increased use of macrolides following NTM acquisition (Table 5) is rarely a component of a multidrug regimen recommended for the treatment of certain mycobacterial species (e.g., *M. absessus*). In addition to their antibacterial properties, macrolides (i.e., azithromycin) are most often chosen for their immunomodulatory activity to improve respiratory function and reduce the frequency of pulmonary exacerbations [21]. Screening for an NTM infection in CF patients prior to the initiation of macrolide therapy should be a universal practice. The increased use of macrolides after NTM acquisition may reflect the clinical decline of these patients, which supports the earlier observation of NTM infections in CF patients reported in a two-year cross-sectional study from Israel [14]. There were low treatment numbers for both of the NTM positive groups based on the ATS/ IDSA guidelines [6]. However, increasing knowledge and awareness to NTM infections has led to different management strategies in recent years [6, 7].

This study has several limitations: 1) NTM screening was not routinely practiced at our center during the 10-year study period; however, it may have been performed more often in patients who did not respond satisfactorily to conventional treatments. As a result, our data may not reflect the overall prevalence of these organisms in the study population; 2) our cohort did not have a large variety of CF gene mutation profiles. This is primarily attributed to the predominance of Caucasians in our patient population, which precluded our ability to detect a relationship between ethnicity or genotype and NTM infections; 3) retrospective studies can be limited by ascertainment bias, despite our best efforts to review every available medical record; and 4) a single, tertiary care, referral center study with small sample size may not adequately represent the entire CF population in the US; multiple variables (e.g., race, geography, and practice patterns) may influence the disease presentation and outcome.

## Conclusion

This single CF center study sheds light on the negative impact of NTM infection in the smaller airways of patients with CF and reveals a significant link between age at diagnosis and NTM infection. Future prospective, multicenter studies with larger and more diverse patient populations are required to better define the impact of NTM on CF outcomes and how infections can best be detected and managed in this population. Thus, our findings indicate that increased awareness by clinicians on different NTM subtypes and more universal treatment plan for NTM infection in the CF population may positively impact patient management and outcomes.

## Abbreviations

ABPA: Allergic bronchopulmonary aspergillosis
CF: Cystic fibrosis
CHW: Wisconsin cystic fibrosis center
FEF25–75: Percent predicted forced expiratory flow at 25–75%
FEV1: Forced expiratory volume in 1 s
MABSC: *Mycobacterium abscessus* complex
MAC: *Mycobacterium avium* complex
NTM: Nontuberculous mycobacteria
